# Clinical Profile, Trends, and Management in Pediatric Patients with Audiovestibular Disorders: Can We Predict Emotional Disability in Pediatric Patients with Episodes of Vertigo and Dizziness?

**DOI:** 10.3390/audiolres14040059

**Published:** 2024-08-14

**Authors:** Joan Lorente-Piera, Nicolás Pérez-Fernández, Melissa Blanco-Pareja, Raquel Manrique-Huarte, Pia Michael Larenas, Valeria Serra, Manuel Manrique

**Affiliations:** 1 Department of Otorhinolaryngology, Clínica Universidad de Navarra, 31008 Pamplona, Spainvaleria.is.serra@gmail.com (V.S.);; 2 Department of Otorhinolaryngology, Clínica Universidad de Navarra, 28027 Madrid, Spain; 3 Department of Otorhinolaryngology, Hospital Clínico de la Universidad de Chile, Santiago 8380453, Chile

**Keywords:** vertigo, pediatric ENT, Ménière, hearing loss, dizziness

## Abstract

Background: Audiovestibular disorders in childhood occur with considerable frequency. However, the difficulty of obtaining medical history, the nonspecificity of symptoms, and the lack of cooperation during complementary tests often contribute significantly to diagnostic biases, attributing clinical presentations to psychosomatic disorders. The objectives of this work are, firstly, to characterize, from an auditory and vestibular perspective, the most frequent causes of vertigo in childhood and a possible relationship with emotional symptoms. On the other hand, to propose the usefulness of the MSSQ-Short questionnaire as a predictive variable in the evolution of children diagnosed with recurrent vertigo of childhood (RVC). Methods: An observational cross-sectional study was designed with retrospective data collection at three tertiary hospitals. Results: Among the 117 patients recruited between 2016 and 2024, 32 patients (27.35%) were diagnosed with an anxious-depressive syndrome prior to audiovestibular testing. The mean age was 11.19 ± 5.61 years and the most frequent final diagnoses were vestibular migraine (VM) with 41.03% and RVC with 23.93%. Patients with VM, compared with RVC, are approximately 1.12 times more likely to have psychosomatic pathology (CI 0.39 to 3.25). The most sensitive and frequently altered test was VEMPS (39.32%), with statistical significance in VM and otic capsule dehiscence, while regarding the MSSQ-Short questionnaire, the linear regression of 0.28 indicates an increase in clinical duration with high questionnaire scores. Conclusions: Vestibular disorders causing dizziness and vertigo are challenging to diagnose, often due to lack of cooperation and/or symptom nonspecificity. A thorough medical history and complementary tests, including audiovestibular and imaging studies, are advisable, thus avoiding systematically attributing children’s complaints to other psychosomatic disorders.

## 1. Introduction

Vertigo is commonly described as the sensation of rotational, intrinsic, or extrinsic oscillation of one’s surroundings or displayed movement [1]. It is a prevalent condition among the younger population, with an estimated prevalence rate ranging from 5.2% to 6.0% in pediatric patients (under 18 years), particularly among females [2]. This symptom can significantly impact children and adolescents, potentially leading to delayed postural control, lack of coordination, and the development of paroxysmal head tilt as a compensatory mechanism for the deficit [3]. While there are common causes of vertigo across age groups, both the etiology and epidemiology of vertigo can vary with age [4]. In pediatric populations, vestibular migraine (VM) and recurrent vertigo of childhood (RVC) are considered the primary causes of episodic vertigo. Conversely, in adults, benign paroxysmal positional vertigo (BPPV) is the most common acute cause, possibly due to anatomical differences that facilitate the fixation of otoconia in the inner ear in children [5]. In adults, unlike young patients, ischemic stroke is another significant chronic cause of vertigo, likely due to the presence of cardiovascular risk factors that are generally absent in childhood. These risk factors play a crucial role in promoting cardiovascular events, potentially leading to infarcts at both the labyrinthine and cerebellar levels [6].

Diagnosing vertigo in children can be challenging due to the variety of causes and the imprecision in symptom descriptions. As early as 1984, Abe et al. [7] suggested that the immaturity of the vestibular system in children could contribute to vestibular disturbances [8]. Additionally, Balatsouras et al. [9] in 2007 pointed out the clinical evaluation challenges, such as anxiety and poor communication skills in children, which can delay audiovestibular testing in cases of vertigo or chronic instability [10].

Our hypothesis is to investigate whether vertigo in children is a consequence of emotional stress and whether these patients consistently exhibit an underlying anxious-depressive component. According to Erbek et al. [11], the relationship is not straightforward; their study on psychogenic vertigo in 2006 revealed a bidirectional relationship between neuro-otological and psychiatric disorders. Often, vestibular or central pathologies manifest before any biopsychosocial symptoms, with the debilitating nature of the condition leading to social withdrawal to avoid triggering stimuli. This isolation can, in turn, result in anxiety, depression, and/or behavioral disorders, contributing to significant emotional stress.

The objectives of this study are twofold: first, to characterize the most frequent causes of vertigo in children from an auditory and vestibular perspective and explore the potential relationship with emotional symptoms; second, to propose the MSSQ-Short questionnaire as a useful predictive tool in assessing the progression of children diagnosed with RVC.

## 2. Materials and Methods

### 2.1. Clinical Study Design

An observational cross-sectional study with retrospective data collection was designed at three tertiary care centers.

### 2.2. Patient Selection

Clinical data were collected from pediatric subjects who presented to the otolaryngology department of our center with symptoms of vertigo or instability from 2016 to 2023 and underwent audiovestibular testing. A detailed review of medical records and relevant tests allowed for the classification of patients with audiovestibular pathology.

### 2.3. Inclusion Criteria

(1) Individuals under 18 years of age presenting to the Otolaryngology department with symptoms of vertigo or instability.
(2) Patients with vestibular and auditory alterations, regardless of the involvement of other structures in the otolaryngological area.
(3) Informed consent was obtained from their legal representatives, who agreed to participate in the study following the 1975 Declaration of Helsinki.

All patients underwent a physical examination based on otoscopy and otoneurological examination, following a correct HINTS protocol (Strupp et al. [12]), with videonystagmography glasses (VideoFrenzel Interacoustics VF505m, MiddleFart, Denmark). Spontaneous nystagmus was registered with the patient seated with their gaze ahead, rightward, and leftward, and in all of them with and without visual fixation. Time for registration was 30 s. Then, intense vibratory stimulus to the skull (SVIN) was performed. We followed a previously used protocol where the SVIN was evoked in a sitting patient by stimulating both mastoid process for 15 s using a 100 Hz handheld vibrator (VVIB 100; Synapsys, France). Nystagmus evoked upon stimulation was recorded using videonystagmoscopy in the dark. Subjects were instructed to continue looking straight ahead while stimulation. SVIN was considered positive when the same nystagmus was registered placing the vibrator in both the right and left mastoids.

For assessing auditory status, tonal audiometry (AC40, Interacustics, MiddleFart, Denmark) was conducted. On the other hand, for vestibular assessment, we used vHIT (GN Otometrics, Taastrup, Denmark) to study the VOR-gain value of the six semicircular canals, both horizontal and vertical, with a normal response considered to be a gain greater than 0.8 in each canal. For the vestibular evoked myogenic potentials (VEMP) tests (Eclipse, Interacoustics, Middlefart, Denmark), including both cervical (cVEMP) and ocular (oVEMP) tests, normal vestibular function is defined as the presence of a vestibular evoked myogenic potential in both ears. This was analyzed using the interaural asymmetry ratio (IAAR (%)) for air-conducted stimulation at 0.5 kHz, 1 kHz, and 4 kHz. The intensity of the acoustic stimulus used was 97 dB HL normalized, and one hundred averages was presented at a rate of 5.1/s. The cVEMP was recorded with patients seated in an upright position. The obtained signals were rectified according to the contraction value of the SCM muscle (sternocleidomastoid muscle). The oVEMP was recorded with the patient seated in an upright position, with their head facing forward and with them being instructed to look at a fixed point on the wall with a 35° upward inclination. Normality is defined as an IAAR of less than 50% in the studied stimulus.

For patients with diagnostic doubts, candidates for cochlear implantation (CI) or suspected associated inner ear malformations, a computed tomography (CT) scan with slices 0.4 mm thick and/or a brain magnetic resonance imaging (MRI) was performed. In patients with suspected endolymphatic hydrops justifying their audiovestibular clinical symptoms, a magnetic resonance imaging (3T) was performed with a T2-FLAIR sequence 4 h after intravenous gadolinium administration to grade cochlear and vestibular endolymphatic hydrops (EH).

It is important to note that all of the patients in the sample, who were evaluated and diagnosed by an otolaryngologist, had previously been assessed by a general pediatrician. The pediatrician was responsible for referring the patient to the appropriate specialist, whether it was a pediatric otolaryngologist or otoneurologist if a vestibular disorder was suspected, or a child psychiatrist if a psychosomatic illness was suspected.

### 2.4. Medical and/or Surgical Treatment

Appropriate medical prescription for each case was based on the latest guidelines and the most recent literature updates [13,14]. Additionally, patients in our cohort had to undergo surgical treatment, either due to the diagnosis of pilocytic astrocytoma of the bulbo-medullary region, complemented with adjuvant radiotherapy, through a posterior fossa craniotomy and partial excision of the exophytic tumor adjacent to the bulbo-medullary dorsum. Patients with severe-profound hearing loss underwent cochlear implant surgery using the standard method with the Cochlear Nucleus CI632 model (Cochlear, Sidney, Australia). For patients with intractable Ménière’s disease (MD), endolymphatic sac decompression surgery was performed by making a decompression below the imaginary line extending from the lateral semicircular canal (Donaldson’s line). Finally, for those with a perilabyrinthine fistula, attempts were made to address it via a retroauricular approach, accessing the oval window and closing the defect with autologous fascia and fibrin glue (Tissuecol; Baxter AG, Wien, Austria).

### 2.5. Follow-Up

All patients underwent follow-up to evaluate the results of medical and surgical interventions, when necessary, through tonal audiometry to measure improvements in mean tone thresholds, vHIT, and VEMPS. For patients receiving maintenance medical treatment and/or surgical treatment, re-evaluation was conducted at three and six months and then annually after completing the protocol.

### 2.6. Measurement via Questionnaires

Only in patients diagnosed with RVC and cinetosis was the Motion Sickness Susceptibility-Short (MSSQ-Short) used, a modification of the original MSSQ, with 9 items aimed at patients under 12 years old, and which was designed to determine the frequency of symptoms and how prone a patient is to develop motion sickness, as well as what types of movement (such as transportation and various activities) are most effective at causing that sensation (Golding et al., 2006 [15,16]). Although the MSSQ-Short is traditionally used to measure the frequency of symptoms, in this study it was used to explore its relationship with symptom duration. This approach allows for the investigation of not only the frequency of motion sickness symptoms, but also how this susceptibility may influence the persistence of symptoms in patients.

### 2.7. Representation and Statistical Analysis

Descriptive statistical methods were employed, primarily arithmetic means, standard deviations, and ranges.

To investigate whether age and vestibular system maturity significantly influence the diagnosis of RVC and VM, an analysis was performed using Student’s *t*-test. Considering that 12 years was chosen as the cutoff point, as it is the upper age limit of the MSSQ-Short questionnaire, and below this age is when there is a higher incidence of RVC and motion sickness [7,8], patients were divided into two groups according to their age: those under 12 years and those aged 12 years or older. T values were calculated for each group, and it was assessed whether there was a statistically significant difference between them. On the other hand, to determine the relationship between the most frequent diagnoses and the presence of spontaneous nystagmus, vibration-induced nystagmus (SVIN), altered vHIT, and VEMPS, Fisher’s exact test was conducted.

Additionally, the Odds Ratio (OR) was calculated to evaluate the strength of the odds of having psychosomatic symptoms in the RVC and VM groups, the two most frequent ones. Likewise, Spearman’s correlation between MSSQ-Short scores and symptom duration was calculated, followed by a simple linear regression model to assess the relationship between these two variables, which was depicted in a scatter plot along with its coefficient of determination (R^2^). The adequacy of the distribution data were checked using the Shapiro–Wilk test to assess the normality of the distribution. For statistical analysis, a significance level of *p* < 0.05 was considered, and RStudio 1.4.1106 was used.

## 3. Results

### 3.1. Population

Out of the 3900 patients treated at our three hospitals from 2016 to 2023 for vestibular disorders who underwent tonal audiometry, vHIT, and VEMPS, 117 patients (3.33%) were pediatric. Their demographic characteristics are summarized in Table 1.

### 3.2. Symptoms and Diagnosis

The main reason why patients visited the otolaryngologist was for vertigo attacks, accounting for up to 56.41% (*n* = 66). The second most frequently described cause was instability, accounting for up to 22.22% (*n* = 26), followed by motion sickness in 11.97% (*n* = 14), headache in 7.69% of patients (*n* = 9), hearing loss in 5.13% of children (*n* = 6), and finally, fluctuating ear fullness in 1 patient (0.85%, *n* = 1).

In a sample of 117 patients, 29.06% (*n* = 34) tested positive for spontaneous nystagmus, while SVIN was observed in 17.09% (*n* = 20) of the patients. Abnormal vHIT results were found in 14.51% (*n* = 17). Additionally, 39.32% (*n* = 46) had abnormal VEMP results, indicating potential vestibular dysfunction. Hearing assessment using pure-tone audiometry (PTA) was conducted on 59 patients (50.43%), with an average hearing threshold of 25.54 ± 3.64 dB, indicating mild hearing loss. Among these, 33.89% (*n* = 20) had pathological values, with 30% of them (*n* = 6) exhibiting severe-to-profound hearing loss. Notably, patients with hydropic ear disease, whether due to autoimmune disease or Meniere’s disease, reported a significant impact from their hearing loss. Quantitatively, the most severe cases involved patients with otic capsule dehiscence, vestibular aqueduct enlargement, or perilymphatic fistulas, who often required implantable devices due to severe-to-profound hearing loss.

**Figure 1:**
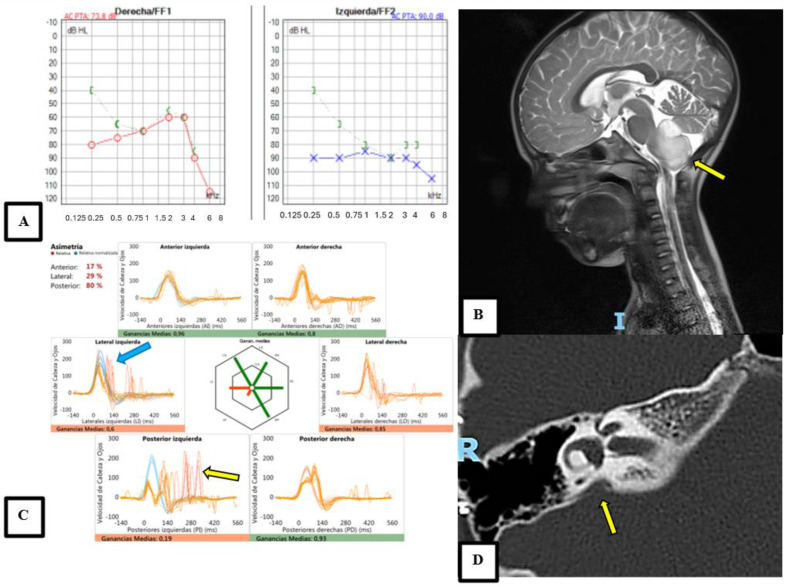
Different complementary tests contributing to the diagnosis of the pathologies included in the sample. (**A**) Shows a pure tone audiometry of a patient with bilateral Ménière’s disease, exhibiting severe hearing loss with an audiogram pattern typical of the disease, with a bone-air gap at low frequencies. (**B**) Depicts the only patient with a brain tumor in the bulbomedullary region (yellow arrow) shown on a sagittal brain MRI. (**C**) Displays a pathological vHIT of a patient with a third window syndrome due to a perilymphatic fistula, showing hypofunctional gains in the left lateral and posterior semicircular canals, with the presence of covert (blue arrow) and overt (yellow arrow) saccades. (**D**) Presents an axial right temporal bone CT scan of one of the patients with vertigo episodes and severe-to-profound hearing loss due to a dilated vestibular aqueduct (yellow arrow).

Complementary imaging tests were performed on 75 patients (64.10%), yielding diagnostic results in 26 patients (34.67%). Based on clinical presentations, audiovestibular test results, and imaging, the following diagnoses were established: vestibular Migraine (VM) was the most common, affecting 41.03% (*n* = 48) of the patients, followed by Recurrent Vertigo of Childhood (RVC), present in 23.93% (*n* = 28) of cases. Idiopathic cases accounted for 7.69% (*n* = 9) and otic capsule dehiscence was observed in 6.84% (*n* = 8). Benign Paroxysmal Positional Vertigo (BPPV) and endolymphatic hydrops were each diagnosed in 5.13% (*n* = 6) of patients. Vestibular paroxysmia and acute peripheral vestibular syndrome were each noted in 3.42% (*n* = 4), while central vertigo was diagnosed in 2.56% (*n* = 3). Post-cochlear implant complications were noted in 0.85% (*n* = 1). Further details about each diagnostic entity are provided in Table 2, with some diagnoses illustrated in Figure 1.

It is noteworthy that all patients were initially screened and evaluated by a general pediatrician to determine whether their symptoms were more psychosomatic, warranting referral to a child psychiatrist, or indicative of a vestibular disorder, leading to an otolaryngologist’s evaluation. Despite this thorough initial screening, 32 patients (27.35%) were diagnosed with a psychosomatic illness by a child psychiatrist before being seen by the otolaryngologist. Moreover, 21 of them (65.63%) were on antidepressant pharmacological treatment and/or concomitant psychotherapeutic treatment. It was ultimately concluded that fourteen of the 48 (29.92%) patients VM had concomitant psychosomatic illness, whereas only 7 of the 26 (26.92%) patients with RVC had it. The odds ratio value is approximately 1.12 with a 95% confidence interval (CI 0.39 to 3.25). This value indicates that children with VM, compared with RVC, are approximately 1.12 times more likely to have psychosomatic pathology, although this result is not statistically significant given the wide confidence interval.

Additionally, when attempting to associate an age under 12 years with the risk of developing RVC, we found that, for a mean age of our sample of 9.24 ± 2.64 years of the patients with RVC, the relationship was statistically significant (*p* < 0.001). In contrast, when associated with MV patients, we observed that, for a mean age of our sample of 11.76 ± 2.73 with this diagnosis, the relationship was not statistically significant (*p* = 0.063).

### 3.3. Questionnaires

**Figure 2:**
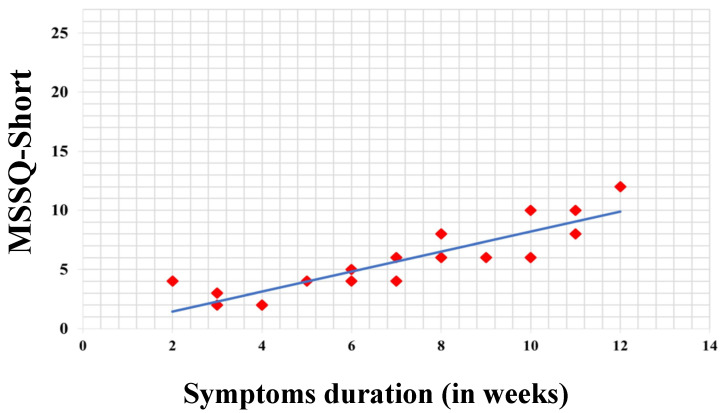
Association of MSSQ-Short questionnaire scores in patients with RVC and symptom duration. The regression coefficient result is 0.28 and the coefficient of determination (R^2^) is 0.78.

The MSSQ-Short questionnaire was administered to 18 of the 28 patients diagnosed with RVC (64.28%), since this diagnosis is particularly indicated in patients under 12 years old, resulting in an average score of 7.00 ± 2.59. In order of frequency, patients reported that dizziness worsened during bus rides, car rides, small boats, and amusement park rides. Spearman’s correlation yielded a result of 0.26, while in linear regression, the coefficient provided a value of 0.28. The coefficient of determination (R^2^) was 0.78, indicating a strong relationship between the two variables in the model’s context, explaining approximately 78.00% of the observed variability in symptom duration based on MSSQ-Short scores (Figure 2).

### 3.4. Treatment

**Figure 3:**
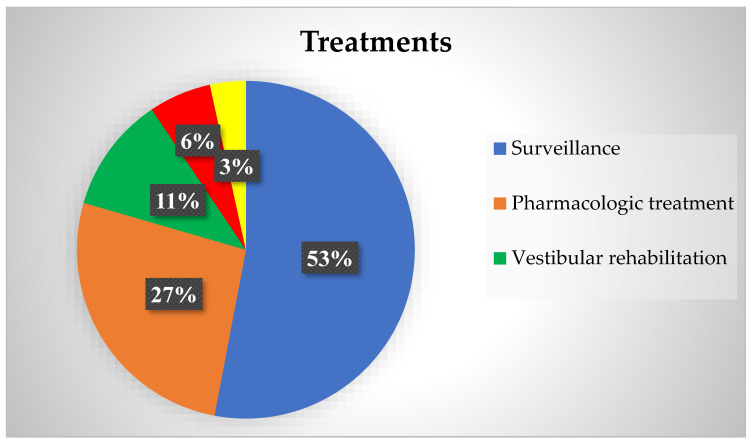
Representation of the distribution of the different treatments used in the patients studied.

Regarding treatment, as depicted in Figure 3, 62 patients (52.99%) were placed under surveillance to monitor their condition and manage symptoms conservatively. Vestibular rehabilitation was prescribed for 13 patients (11.11%) to help improve balance and reduce dizziness through specific exercises. Calcium channel blockers were given to ten patients (8.55%) as part of their management plan. Antihistamines were recommended to two patients (1.71%) to help alleviate symptoms of dizziness and nausea.

Repositioning maneuvers, such as the Epley and Lempert maneuvers, were performed on four patients (3.42%) to BPPV. Oxcarbazepine was used by two patients (1.71%) to manage symptoms with vestibular paroxysmia. Triptans were prescribed for five patients (4.27%) to treat migraine-associated vertigo. Acetazolamide was given to four patients (3.42%) to reduce intracranial pressure and manage associated symptoms.

Surgery was performed on seven patients (5.98%). Of these seven, six underwent cochlear implantation surgery, with four receiving unilateral implants and two receiving bilateral implants. During the surgery, in one of them, a perilymphatic fistula closure was performed using a retroauricular approach, while in another one, endolymphatic sac decompression was performed simultaneous to the cochlear implantation procedure to treat an MD with a sluggish response to medical treatment. The remaining patient underwent surgery to remove a brain tumor. Tryptizol was used by two patients (1.71%) for its antidepressant and pain-modulating effects. Natalizumab was prescribed for one patient (0.85%) with remittent recurrent multiple sclerosis. Topiramate was used by one patient (0.85%) to help prevent migraines and reduce vertigo symptoms. Radiotherapy was employed for one patient (0.85%) with astrocytoma together with surgery. Sulpiride was used by one patient (0.85%) to manage dizziness and associated symptoms with a multicanal BPPV.

The total number of treatments used is summarized in Figure 3. Additionally, Table 3 specifically describes the approach to treating each of the diagnoses in our cohort.

## 4. Discussion

Vestibular pathology in childhood and adolescence can present with a highly heterogeneous range of symptoms, as shown in Table 2, indicating that the resulting diagnoses are also very diverse [17]. Infants and young children under 2 years old appear to be relatively protected against RVC. After this age, susceptibility increases, peaking between 7 and 12 years, and then gradually decreases [18,19]. This pattern aligns with our sample, where the mean age was 11 years, demonstrating a statistically significant association between age and diagnoses, such as RVC, one of the most frequent conditions in the cohort (*p* < 0.001). Additionally, in 2006, Golding et al. [20] observed a higher tendency for these disorders to develop in females, particularly around the menstrual cycle [21].

Regarding diagnosis, the literature indicates that VM is the most common cause of vertigo and instability in childhood, which is consistent with our study’s findings [22]. Although the pathophysiology of VM is not fully understood, current hypotheses suggest a connection to migraines, highlighting the role of trigeminal innervation in labyrinthine vessels and the presence of vasoactive neuropeptides in perivascular terminals of trigeminal fibers (Espinosa-Sánchez et al. [23]). This prevalent condition is influenced by genetic, epigenetic, and environmental factors, with increased sensitivity to emetic stimuli, especially in adolescent females, as observed in our sample, where there was a clear predominance of the female gender [24,25]. Although the American Academy of Neurology [26] has not established a consensus on prophylactic treatment for VM crises, there is evidence supporting a positive response to flunarizine [27], triptans, or diuretics like acetazolamide [28]. However, preventive treatment is generally recommended only when the frequency of episodes or symptoms significantly affects daily activities or neurodevelopment. Consequently, active surveillance was employed in up to 66% of patients with VM.

In our analysis, we particularly emphasize the entity known as RVC. Although its exact origin is unknown, it is believed to result from a vestibulo-visual dissonance where disturbances in the visual system, spatial orientation signals, and difficulty integrating multisensory information lead to imbalance and vertigo [29]. Disorders such as MV and MD are associated with higher susceptibility to motion sickness [30]. Regarding the MSSQ-Short questionnaire, we observed similarities in average scores compared to the literature (Golding et al. 2006 [16]), with a particular tendency to trigger symptoms in small vehicles and buses, consistent with our cohort. Moreover, as proposed in our objectives, although the Spearman correlation coefficient for our sample was 0.26, indicating a weak relationship, the linear regression model provided a coefficient of 0.28, suggesting that an increase in MSSQ-Short scores is weakly associated with an increase in symptom duration. Thus, the questionnaire could have valuable predictive potential, warranting further assessment in larger samples of children under 12 years with RVC.

Among interesting otoneurological findings from the study, recent years have seen increased emphasis on the importance of using SVIN and its connection to various vestibular system pathologies [31]. When SVIN is present, it indicates a functional asymmetry between the vestibular nuclei on the right and left sides, primarily originating at the labyrinth level [32]. Consequently, SVIN is considered to be a useful bedside test for patients with vertigo, dizziness, and other vestibular symptoms. In patients with unilateral vestibular loss, a 100 Hz bone-conducted vibration applied to either mastoid induces a predominantly horizontal nystagmus with fast phases beating away from the affected side. This phenomenon is particularly relevant in patients with recurring vertigo and migraine symptoms, as those who predominantly exhibit SVIN are more prone to developing MD [33]. We observed this finding in 20 patients, notably in those with hydropic inner ear disease, where up to 75% showed this response during vibration induction.

Another condition gaining prominence, with increased focus on characterization and new anatomical-clinical classifications [34] to better understand its pathophysiology, is otic capsule dehiscence. In our sample, it was the fourth most frequent condition, accounting for up to 7% of cases. Our findings align with studies like Dasgupta et al. in 2020 [35], which identify superior semicircular canal dehiscence and enlarged vestibular aqueduct as the most common third window syndromes in pediatric patients. This study also highlights the utility of VEMPs in diagnosing these conditions, with pathological VEMP results observed in 100% of our dehiscence cases.

Finally, it is crucial to note that MD is exceptionally rare in childhood, with a prevalence ranging from 0.4% to 5% [36,37]. Although the course of MD can vary, an aggressive progression has been observed in pediatric cases, often involving significant auditory impairment and increased endolymphatic hydrops. Another significant contribution of our study is confirming the use of VEMPs for differentiating between VM and MD. As suggested by Dlugaiczyk et al. [38], cVEMP amplitude may significantly differ between these groups, with VM patients typically presenting decreased amplitudes and MD patients showing increased responses at 1000 Hz, possibly due to unilateral temporal hypoperfusion of the labyrinthine artery.

Regarding the last two conditions described, both MD and otic capsule dehiscence, it is intriguing to explore why these conditions can lead to a significant emotional impact. For MD, whose etiology remains unclear, factors such as seasonal changes, meteorological conditions, or stress can disrupt inner ear homeostasis, exacerbating symptoms [39]. Patients often avoid situations that might trigger auditory fluctuations or vertigo attacks. On the other hand, otic capsule dehiscences, causing audiovestibular symptoms due to alterations in fluid dynamics from the absence of bony cortex [40], can also lead patients to avoid adverse situations. Specifically, many of these patients may isolate themselves to avoid symptoms like Tullio’s phenomenon (dizziness induced by noise) or Hennebert’s sign (dizziness with barometric changes), thereby trying to prevent marked symptomatic episodes [41]. Thus, in both cases, there is often an environment of isolation and limited social contact, potentially contributing to the development of anxiety and depressive symptoms.

In summary, the principal limitation of our study is the follow-up period. Although extended follow-up was not the primary focus of our study, which aimed at better characterization, it would be beneficial to observe patients for six months to a year. This would allow us to assess how patients respond to treatment and whether improvements in vestibular symptoms correlate with improvements in psychosomatic symptoms.

Another significant limitation we encountered was assessing emotional disability and quality of life in our patients. Given that these are pediatric patients, adherence to completing extensive questionnaires, commonly used in vertigo studies, was limited. The lack of cooperation often made it challenging to evaluate aspects like gait or conduct dynamic tests using other types of questionnaires. However, brief and less demanding questionnaires, such as the MSSQ-Short, have provided valuable insights into RVC. Therefore, it would be beneficial to introduce or adapt other questionnaires that allow for better adherence, helping to reflect changes in quality of life for this age group.

## 5. Conclusions

The difficulties in diagnosing childhood vertigo, due to the peculiarities mentioned involving the pediatric population, often lead to attributing all symptoms to psychosomatic disorders and not to their most common causes, such as MV or BVPI. Frequently, the disability they generate in the development of the patient’s daily activities produces emotional stress that can mask an underlying vestibular disorder or central pathology that has medical or even surgical treatment. Both our sample and the literature indicate that between 30 and 50% of these patients may present pathological otoneurological findings and/or audiovestibular test abnormalities, so these tests should never be stopped in order not to overlook entities, which, once treated properly, can have a minimal impact on the patients’ quality of life. The disability caused by emotional stress can interfere with daily activities and is perceived to be one of the most important aspects of vestibular dysfunction, highlighting the need for education among pediatric providers.

## Figures and Tables

**Table 1:** Demographic characteristics of the studied patients.

Demographic Description		
Age At Diagnosis	11.19 ± 5.61 (1–18) years	
Genre	70 (59.82%) Women	47 (40.17%) Men
Follow-Up	4.33 ± 1.29 (1 month–6.23 years)	

**Table 2:** Summary of the diagnoses found in our sample, as well as the results of tests and vestibular findings. SSCDS: Superior Semicircular Canal Dehiscense Syndrome; EVA: Enlarged Vestibular Acueduct; HSC: Horizontal Semicircular Canal; PSC: Posterior Semicircular Canal; MD: Ménière Disease; VP: Vestibular Paroxysmia.

Diagnostic	Patients	Spontaneous Nystagmus	SVIN	Altered vHIT	Altered VEMPS	Other
VM	48 (41.03%)	*n* = 6 *p* value = 0.078	*n* = 4 *p* value = 0.739	*n* = 4 *p* value = 1.000	*n* = 14 *p* value = 0.512	*n* = 15 Definitive *n* = 33 Probable VM
RVC	28 (23.93%)	*n* = 3 *p* value = 1.00	*n* = 3 *p* value = 1.00	*n* = 1 *p* value = 1.00	*n* = 7 *p* value = 1.00	-
Idiopathic	9 (6.84%)	*n* = 0 *p* value = 0.571	*n* = 0 *p* value = 0.529	*n* = 0 *p* value = 1.00	*n* = 0 *p* value = 1.00	-
Otic capsule dehiscence	8 (6.84%)	*n* = 3 *p* value = 0.072	*n* = 4 *p* value = 0.051	*n* = 4 *p* = 0.009	*n* = 8 *p* value ≤ 0.001	*n* = 4 SSCDS *n* = 2 EVA *n* = 1 Perilymphatic fistula and 1 PSC
BPPV	6 (5.13%)	*n* = 0 *p* value = 1.00	*n* = 1 *p* value = 1.00	*n* = 0 *p* value = 1.00	*n* = 2 *p* value = 1.00	*n* = 2 HSC and 2 PSC *n* = 2 Multicanal
Endolymphatic hydrops	6 (5.13%)	*n* = 3 *p* value = 0.072	*n* = 3 *p* value = 0.153	*n* = 3 *p* value = 0.153	*n* = 6 *p* value ≤ 0.001	*n* = 3 Autoimmune ear disease *n* = 3 definitive MD
Acute vestibular syndrome	4 (3.42%)	*n* = 4 *p* = 0.072	*n* = 2 *p* value = 0.003	*n* = 3 *p* value = 0.003	*n* = 3 *p* value = 0.194	*n* = 2 Vestibular neuritis *n* = 2 Laberinthitis
Vestibular paroxysmia	4 (3.42%)	*n* = 2 *p* = 0.001	*n* = 0 *p* value = 0.286	*n* = 0 *p* value = 1.00	*n* = 2 *p* value = 1.00	*n* = 2 Definitive and 2 Probable VP
Central vertigo	3 (2.56%)	*n* = 2 *p* value = 0.070	*n* = 2 *p* value = 0.076	*n* = 2 *p* value = 0.286	*n* = 3 *p* value = 1.00	*n* = 1 Multiple sclerosis *n* = 1 Pilocytic astrocytoma *n* = 1 Rathke cleft cyst
Post cochear implantation	1 (0.85%)	*n* = 1 *p* value = 0.019	*n* = 1 *p* value = 0.033	*n* = 0 *p* value = 0.033	*n* = 1 *p* value = 1.00	-

**Table 3:** Summary of the treatments used to address each of the symptoms derived from the diagnoses of the patients in our sample. IH: Intracranial hypertension.

Diagnostic	Treatment
Vestibular migraine	Surveillance = 32 Calcium channel blockers = 6 Triptans = 5 Acetazolamide = 2 Tryptizol = 2 Topiramate = 1
RVC	Surveillance = 17 Vestibular rehabilitation = 7 Antihistamines = 2 Calcium channel blockers = 2
Idiopathic	Surveillance = 5 Vestibular rehabilitation = 4
Otic capsule dehiscence	Surveillance = 4 Surgery for cochlear implantation = 3 Acetazolamide = 1
BPPV	Lempert maneuver = 2 Epley maneuver = 2 Sulpiride = 1 Surveillance = 1
Endolymphatic hydrops	Surgery for cochlear implantation = 3 Surveillance = 2 Acetazolamide = 1
Acute vestibular syndrome	Steroids = 2 Vestibular rehabilitation = 1 Surveillance = 1
Vestibular paroxysmia	Oxcarbazepine = 2 Calcium channel blockers = 2
Central vertigo	Surgery and radiotherapy for astrocytoma = 1 Azetazolamide for IH due to Rathke cyst = 1 Natalizumab for multiple sclerosis = 1
Post cochear implantation	Vestibular rehabilitation = 1

## Data Availability

Data pertaining to this study can be shared upon request to the corresponding author.
